# Bilateral breast lymphoma in lactating woman: rare and particularly aggressive entity

**DOI:** 10.1093/omcr/omaf014

**Published:** 2025-03-28

**Authors:** Fatima Ezzahra Lahlimi, Ouadii Abakarim, Ibtissam Mhirig, Illias Tazi

**Affiliations:** Department of Clinical Hematology & Bone Marrow Transplantation, University Hospital Center Mohammed VI, Faculty of Medicine and Pharmacy, Cadi Ayyad University, Marrakesh, Morocco; Department of Clinical Hematology & Bone Marrow Transplantation, University Hospital Center Mohammed VI, Faculty of Medicine and Pharmacy, Cadi Ayyad University, Marrakesh, Morocco; Department of Biology, University Hospital Center Mohammed VI, Faculty of Medicine and Pharmacy, Cadi Ayyad University, Marrakesh, Morocco; Department of Clinical Hematology & Bone Marrow Transplantation, University Hospital Center Mohammed VI, Faculty of Medicine and Pharmacy, Cadi Ayyad University, Marrakesh, Morocco

**Keywords:** breast lymphoma, DLBCL NonGCB, aggressive presentation, poor prognosis, case report

## Abstract

Non-Hodgkin’s lymphoma (NHL) is seldom encountered in breast. Primary malignant lymphomas of the breast in pregnant or lactating women are more uncommon with massive bilateral breast enlargement rapidly followed by widespread dissemination to several organs. Its diagnosis is often delayed, its treatment still be defined, and its prognosis is particularly poor. We report the case of a 20-year-old Moroccan woman with bilateral primary diffuse B-cell lymphoma of the breast, treated with chemotherapy without a favorable response, progressed to deterioration then death. This case highlights the management difficulties of this group of very high-risk lymphomas.

## Introduction

Primary breast lymphomas (PBL) are defined as malignant lymphomas primarily affecting the breast, with or without regional lymph node involvement, and in the absence of other sites [1]. PBL are rare, accounting for less than 1% of all non-Hodgkin’s lymphomas and about 2% of extra nodal locations. Similarly, they constitute less than 1% of all breast tumors [2]. Several studies have differentiated between two distinct groups of primary breast lymphoma: the first group is predominantly unilateral, B-phenotype and affects women between 60 and 65 years of age, and the second group is rarer, often bilateral with a younger age of onset, histological burkitt or burkitt-like [2–4]. This second group is characterized by a disseminated, aggressive, and often fatal presentation and has been frequently associated with pregnancy and lactation [3].

We report the case of a young Moroccan patient with a primary breast lymphoma fitting the second group described, we want to highlight the difficulties of management of this rare entity and very poor prognosis.

## Case report

A 20-year-old woman, married and breastfeeding for 3 months, without any past history, including malignancies, presented with bilateral breast nodules, increasing volume, and painful tension, prompting breastfeeding cessation. She reported no B-symptoms. Duration of evolution was 2 months.

On examination of the right breast, a gigantomastia was found with slight nipple retraction and a 15 cm mass along with 4 cm homolateral axillary adenopathy. The contralateral breast exhibited a 4 cm mass without lymphadenopathy.

Mammography depicted dense breasts with increased density, devoid of architectural alterations or microcalcifications. A large, relatively well-limited watery opacity occupied a significant portion of the right breast mass (measuring 120/80 mm). Ultrasound confirmed infectious mastitis on the right and a fibroadenoma-like appearance on the left breast.

**Figure 1:**
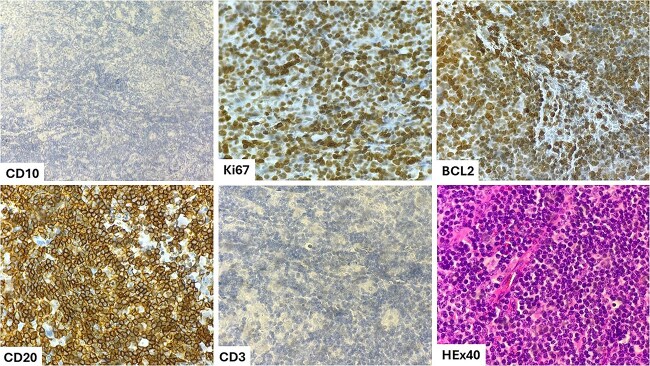
Malignant proliferation of large round cells, CD3−, CD10−, CD20+, BCL2+, Ki67+.

Histological examination revealed an undifferentiated tumor proliferation with large elements showing sparse cytoplasm and irregular, nucleated nuclei with abnormal mitoses. The cells were negative for anti-cytokeratin, CD3, and CD10 antibodies but positive for CD20, bcl2, and MUM1 antibodies, indicating a diffuse large B-Cell malignant lymphoma (DLBCL) with a non-germinal center phenotype (Fig. 1).

Extension work-up, including thoracoabdominal CT and bone marrow biopsy, showed no abnormalities, confirming a stage IIE lymphoma according to the Ann Arbor classification.

**Figure 2:**
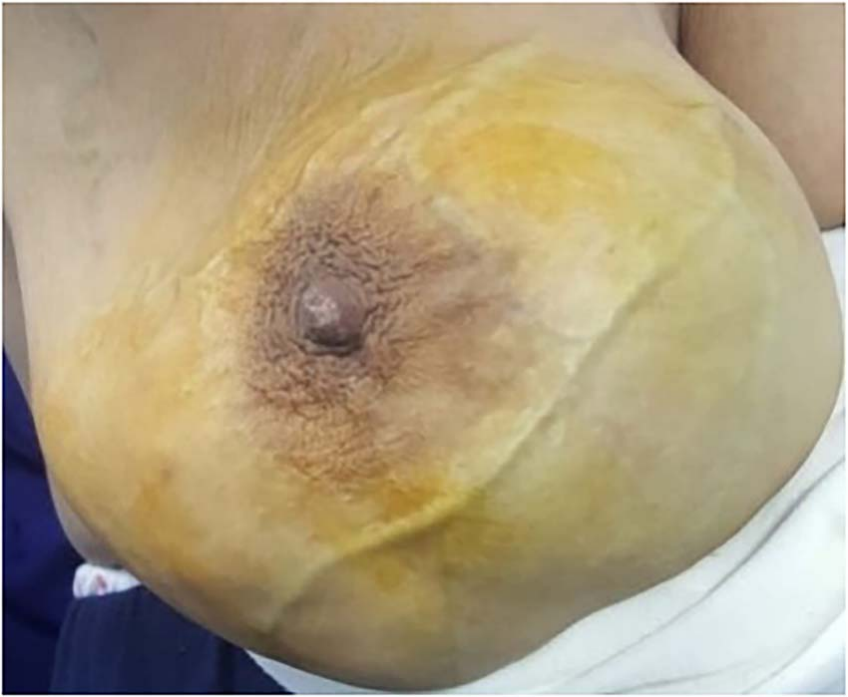
The right breast tumor after progression under treatment.

Serological tests for hepatitis B, hepatitis C, cytomegalovirus, and human immunodeficiency viruses (HIV) 1 and 2 were negative. The patient received Rituximab, Cyclophosphamide, Doxorubicin, Vincristine and Prednisone (RCHOP) chemotherapy but showed no clinical improvement after 4 cycles (Fig. 2). CT scan revealed tumor progression with dissemination to the liver, kidneys, bone, peritoneum, and adenopathy. Subsequently, the patient underwent second-line Rituximab, Ifosfamide, Carboplatin and Etoposide (R-ICE) protocol treatment for 2 cycles.

**Figure 3:**
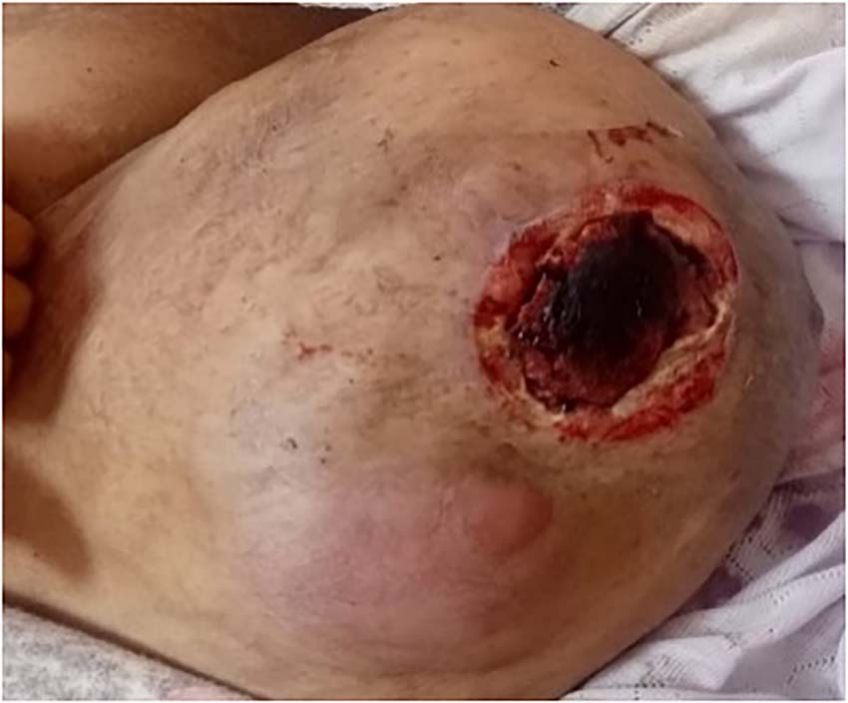
Right breast tumor after 2nd line treatment.

Gigantomastia progressed rapidly, leading to cutaneous fistula and tissue loss amidst deteriorating general health (Fig. 3). Microbiological tests were negative, and a second biopsy confirmed DLBCL diagnosis. Symptoms worsened with headaches and visual disturbances, indicating diffuse central nervous system (CNS) lymphomatous involvement on cerebral MRI. The patient passed away four months post-diagnosis.

## Discussion

PBL are rare. Their frequency is estimated to be 0.04%–0.52% of all breast neoplasia and 2.2% of extra-nodal lymphomas. This entity usually affects women; however, men’s cases have been reported [2, 3].

Breastfeeding may lower breast cancer risk via hormonal shifts and immune benefits, but its specific impact on lymphoma, including breast lymphoma, is uncertain [3].

Authors distinguish two entities of PBL: a common unilateral type, nearly 90% in those aged 60–65, with nonspecific clinico-radiological profiles. Dominated by B phenotype, histologically, DLBCL ranks highest, followed by MALT lymphoma. Treatment involves immunochemotherapy and radiotherapy, showing better control in early stages [4].

The second entity, to which our patient belongs, is bilateral and less common, comprising around 10% of PBL cases [1]. Typically associated with pregnancy or breastfeeding, it affects younger patients, with a median age of 20–30 years [3, 5].

Both entities typically manifest through the development of breast tumors, often accompanied by unilateral or bilateral gigantomastia and inflammatory mastitis, as observed in our patient. Axillary lymph nodes are involved in 20 to 40% of cases [4, 5].

Radiological presentation is nonspecific; mammography often shows benign masses like cysts, fibroadenomas, or phyllodes tumors, occasionally resembling mastitis [6].

The second entity of PBL associated with pregnancy exhibits aggressive presentation, as seen in our case. Diagnostic delays are common, leading to advanced disease stages in over half of patients, worsening prognosis. Dissemination commonly involves multiple organs [1, 4, 5].

Histologically, this group of lymphomas is often the Burkitt’s or Burkitt-like type; some cases of DLBCL have also been reported with high proliferation indexes [2, 4].

Treatment for primary breast lymphoma lacks standardization due to its rarity. CHOP-type protocols often prove insufficient, leading to advocacy for more intensive regimens like CODOX-M/IVAC, with uncertain roles for radiotherapy. Inositol and Boswellia supplementation may reduce breast density [5, 6]. The introduction of a more intensive chemotherapy protocol combined with radiotherapy is being considered to control the severe evolution of this disease [7]. The role of surgery is still being discussed and several authors are against this therapeutic approach [8]. Due to rapid and aggressive evolution to stage IV in our patient with multi-organ involvement, the surgical indication was not considered, and chemotherapy was performed as usual. The following table summarizes literature review of this rare entity (Table 1).

**Table 1:** Literature review of PBL.

Authors/Study	Number of Cases	Histopathological type	Treatment	Outcome	Follow-Up Duration
Hosein et al.	76	DLBCL	Rituximab, Chemotherapy (R-CHOP, Hyper CVAD)	5-year OS rate: 60%, 5-year PFS rate: 52%	5 years
Montilla et al.	1	PBL	R-CHOP	Complete Remission	1 year
Avilés et al.	1	PBL	R-CEEP	Not specified	Not specified
Neri et al.	1	Non-Hodgkin’s Breast Lymphoma	R-CHOP plus Surgery	Successful outcome	Not specified
Georgountzos et al.	1 (HIV-infected patient)	Secondary Breast Lymphoma	Chemotherapy (not specified)	Not specified	Not specified

DLBCL: diffuse large B-Cell malignant lymphoma; PBL: primary breast lymphomas; HIV: human immunodeficiency virus; OS: overall survival; PFS: progression free survival; R-CHOP: rituximab cyclophosphamide doxorubicin vincristine prednisolone; R-CEEP: rituximab cyclophosphamide epirubicin vincristine prednisolone.

Breast lymphoma’s association with human immunodeficiency virus (HIV) is seldom reported [9]. Despite its rarity, preventive measures in pregnant women are advisable to prevent HIV transmission. Dolutegravir-based therapy is effective, alongside atazanavir and lopinavir [10].

The prognosis is particularly poor with 2-year survival rates not exceeding 15% in most small published series [5].

Primary breast lymphoma associated with pregnancy and breastfeeding is a very rare entity with a very poor prognosis. Any atypical breast symptoms in pregnant or breastfeeding women should alert the practitioner to avoid diagnostic delay, more intensive protocols should be evaluated in this high-risk group of patients to improve their prognosis.
